# O-30 - A LOCAL OUTBREAK OF IATROGENIC BOTULISM ASSOCIATED WITH COSMETIC INJECTIONS OF BOTULINUM NEUROTOXIN-CONTAINING PRODUCTS, ENGLAND, 2025

**DOI:** 10.1093/pubmed/fdag046.026

**Published:** 2026-07-09

**Authors:** Mr Joe Jasperse, Kate Wilson, Sana Akbar, Iain Hayden, Qudsia Naseem, Amii Coglan, Matt Hewson, Alan Young, Min Fang, Yvonne Liu, Joanne Darke, Vanessa Wong, Gauri Godbole, Gareth J Hughes

**Affiliations:** Field Service North East and Yorkshire and Humber, UK Health Security Agency; UK Field Epidemiology Training Programme, UK Health Security Agency; North East Health Protection Team, UK Health Security Agency; Field Service North East and Yorkshire and Humber, UK Health Security Agency; County Durham and Darlington NHS Foundation Trust; Gastrointestinal Infections, Food Safety and One Health Division, UK Health Security Agency; County Durham and Darlington NHS Foundation Trust; Durham County Council; Criminal Enforcement Unit, Medicines and Healthcare products Regulatory Agency; Criminal Enforcement Unit, Medicines and Healthcare products Regulatory Agency; Molecular Analysis, Analytical and Biological Sciences, Science and Research, Medicines and Healthcare products Regulatory Agency; Bacterial Vaccine Standards, Standards Lifecycle, Science and Research, Medicines and Healthcare products Regulatory Agency; North East Health Protection Team, UK Health Security Agency; Gastrointestinal Infections, Food Safety and One Health Division, UK Health Security Agency; Gastrointestinal Infections, Food Safety and One Health Division, UK Health Security Agency; Field Service North East and Yorkshire and Humber, UK Health Security Agency

## Abstract

**Background:**

Iatrogenic botulism is a rare but serious illness caused by the systemic spread of an excessive or improperly administered dose of botulinum neurotoxin (BoNT) during medical or cosmetic procedures. Here we report the investigation of an outbreak of iatrogenic botulism linked to cosmetic injections in North East England.

**Objectives:**

To identify risk factors for illness and inform recommendations for minimising future public health risk.

**Methods:**

We defined a case as an individual with a clinical diagnosis of botulism who received a cosmetic injection with a BoNT-containing product from a practitioner in North East England since 1 May 2025 and developed at least one compatible symptom (difficulty swallowing, difficulty breathing, altered speech) within four weeks. We conducted a case-control study with controls recruited via social media and used univariable Firth logistic regression to analyse potential risk factors. An endopeptidase immunoassay was used to estimate the potency of a vial of unlicensed product seized from a local supplier.

**Results:**

Overall, 25 cases were identified with onset dates ranging from 18 May to 9 June 2025. Most cases were female (88%) and the median age was 43 years (range: 25-82 years). Cases were significantly more likely to have attended two specific local practitioners (both p<0.001), reported receiving an unlicensed product (p < 0.001) and received more injections than at their previous appointment (p = 0.029). The estimated potency of the seized unlicensed product (370 units/vial, 95% CI: 327–419 units) was 85% higher than that listed on its labelling (200 units/vial).

**Conclusions:**

Stronger regulation of cosmetic practitioners should be introduced in the UK, where no mandatory training or licensing requirements are currently in place despite rising demand for cosmetic procedures. Raising public awareness around the importance of using licensed products, as well as disrupting the supply of unlicensed products, will help further reduce public health risk.

